# The magnitude of extended-spectrum beta-lactamase- producing *Enterobacteriaceae* from clinical samples in Ethiopia: a systematic review and meta-analysis

**DOI:** 10.1099/acmi.0.000195

**Published:** 2021-01-28

**Authors:** Kuma Diriba, Ephrem Awulachew, Aschelew Gemede, Asrat Anja

**Affiliations:** ^1^​ Department of Medical Laboratory Sciences, Health Science and Medical College, Dilla University, Dilla, Ethiopia

**Keywords:** extended-spectrum β-lactamase, β-lactam resistance, antibiotic resistance, *Enterobacteriaceae*, *Klepsiella pneumonia*, *Escherichia coli*, Ethiopia

## Abstract

**Background:**

The rapid spread of resistance among extended-spectrum β-lactamase (ESBL)-producing *Enterobacteriaceae* is a serious problem around the world. It results in serious clinical complications in humans and has become a global threat. Therefore, this systematic review and meta-analysis was aimed to estimate the pooled prevalence of ESBL-producing *Enterobacteriaceae* in different clinical samples in Ethiopia.

**Methods:**

A systematic search was conducted on PubMed, Web of Science, Embase, Google Scholar and the Cochrane Library. All identified observational studies reporting the prevalence of ESBL-producing *Enterobacteriaceae* from clinical samples in Ethiopia were included. Four authors independently extracted data and analysed using R software version 3.6.1 and STATA statistical software version 13. A random-effects model was computed to estimate the pooled prevalence of ESBL-producing *Enterobacteriaceae* in Ethiopia.

**Results:**

Of 142 articles reviewed, 14 studies that fulfilled the inclusion criteria were included in the meta-analysis. The pooled prevalence of ESBL-producing *Enterobacteriaceae* in the different clinical specimens in Ethiopia was 49 % (95 % CI: 39, 60). *Klebsiella pneumoniae* was the leading ESBL-producing *Enterobacteriaceae* followed by *Escherichia coli* and *Acinetobacter baumannii* with a prevalence of 74, 67 and 60 %, respectively. ESBL-producing isolates showed a high rate of resistance to cefotaxime, ceftriaxone, ceftazidime, Amoxicillin clavulanic acid (AMC), ampicillin and aztreonam. The better options for the treatment of ESBL-producing *Enterobacteriaceae* are amikacin and Imipenem.

**Conclusion:**

The magnitude of ESBL-producing *Enterobacteriaceae* in different clinical samples in Ethiopia is alarmingly high and represents a threat to human health. Hence, a coordinated effort needs to be implemented for the prevention and control of these *Enterobacteriaceae *.

## Background

*Enterobacteriaceae* are a huge, heterogeneous group of gram-negative rods whose natural habitat is the intestinal tract of humans and animals [1]. These microorganisms have emerged as one of the most important reasons for nosocomial and community-obtained infections [2–5]. *Enterobacteriaceae* are typically associated with a range of infections [6], among which urinary tract infections, bloodstream infections, heath facility-associated pneumonia and some intra-abdominal infections are the most crucial [7, 8]. Studies conducted in different underdeveloped countries indicate a high case fatality rate associated with bloodstream infection, due to *Enterobacteriaceae* [9, 10].

Antibiotics play a vital role in decreasing the load of communicable diseases worldwide [11]. Microbial resistance to antimicrobial agents is rising remarkably worldwide [12–14]. This rapid spread of resistance among pathogenic microorganisms is a serious problem globally [2, 4], because it limits drug treatment against infections [15]. Antimicrobial resistance has been recognized as one of the most important problems facing human health by the World Health Organization (WHO) [15, 16]. Frequent isolation of multidrug resistant (MDR) pathogens in both hospital and community-acquired infections has further intensified the problem of antimicrobial resistance [5].

Currently, extended-spectrum β-lactamase (ESBL)-producing *Enterobacteriaceae* represent a serious public health issue globally [1]. They become resistant to beta-lactam antibiotics via the production of beta-lactamase enzymes that inactivate beta-lactam antibiotics, and this continues to be the prominent cause of β-lactam antibiotic resistance among *Enterobacteriaceae* [17, 18]. They can rapidly develop resistance against a range of important broad-spectrum antimicrobials [19, 20]. Inappropriate and irrational use of antimicrobial drugs, and poor sanitary and infection control practices in the area may play a critical role in the increased prevalence of resistant bacteria in a community, providing favourable conditions for resistant microorganisms to emerge and spread [21, 22]. This can lead to a proliferation of organisms with broad-spectrum β-lactamase activity that threatens the future of the β-lactam class in clinical care [21].

The increasing rate of human infections caused by antimicrobial resistance strains of *Enterobacteriaceae* makes clinical management more difficult by prolonging the illness and compromising treatment [5]. This can have a potentially serious impact on human health. The situation is more common in developing countries where there is widespread and uncontrolled use of antibiotics [16]. Data on ESBL-producing *Enterobacteriaceae* in Ethiopia are limited and are not currently available in aggregate form. Therefore, this systematic review and meta-analysis aimed to determine the pooled prevalence of ESBL-producing *Enterobacteriaceae* using available studies in Ethiopia.

## Methods

### Study design

A systematic review and meta-analysis was conducted to estimate the prevalence of ESBL-producing *Enterobacteriaceae* from clinical samples from patients attending health institutions in Ethiopia following the methodological framework suggested by Arksey and O’Malley [23].

### Search strategies

All relevant articles were searched without date limits using the following databases: PubMed, Web of Science, Embase, Google Scholar, Cochrane Library and Science Direct according to the Preferred Reporting Items for Systematic Reviews and Meta-analysis (PRISMA) [24]. All searches were limited to articles written in English given that such language restriction does not alter the outcome of the systematic reviews and meta-analysis [25]. Grey literature of observational studies was searched through the review of reference lists and input of content experts. The search of the literature was conducted from May 2005 to September 2019. All papers published until the end of 2019 that fulfilled the inclusion criteria were considered. The search used the following keywords: ‘Extended-spectrum β-lactamase; β-lactams resistance; antibiotic resistance; Enterobacteriaceae; K. pneumoniae; E. coli; Ethiopia’. We searched all terms with the help of Boolean operators such as ‘AND’ or ‘OR’.

### Eligibility criteria

Studies conducted in Ethiopia and articles reported in English were considered. Only studies involving humans and published articles were included. All observational study designs reporting data regarding the proportion of ESBL-producing *Enterobacteriaceae* isolated from humans were eligible for this review. Studies with the following characteristics were excluded from the analysis: review articles, letters, articles that had no original data, articles that did not identify the species and the origin of isolates, articles that were not fully accessible, performed outside Ethiopia, duplicate publications of the same study and studies involving animals.

### Assessment of study quality

Studies selected for inclusion were assessed for methodological quality by all authors independently using the standard critical appraisal instruments of the Joanna Briggs Institute Meta-Analysis of Statistics Assessment for Review Instrument (JBI-MAStARI) [26]. Disagreements were resolved by consensus

### Outcome measures

The primary outcome variable of this study was the prevalence of ESBL-producing *Enterobacteriaceae *, while the secondary outcome was the antibiotics resistance profile of ESBL-producing *Enterobacteriaceae *.

### Data extraction

The data were extracted using a standardized data extraction format, adapted from the Joanna Briggs Institute (JBI), by three authors (Kuma Diriba, Asrat Anja and Ephrem Awulachew) independently extracting all necessary data. The extracted data were then merged for systematic analysis. Any disagreements during the data extraction were resolved through discussion and consensus. The main outcomes extracted from the study were: primary author, publication year, study method, study area, sample size and cases. Data on associated risk factors were also extracted by the authors.

### Statistical analysis

Following data extraction, systematic review and meta-analysis were carried out using R software version 3.6.1 and STATA statistical software (version 13) with user-contributed commands for meta-analyses: metaprop, metan, metainf, metabias and metareg [27]. The effect sizes and SEs of the studies were pooled using a random-effects model to calculate the pooled prevalence of ESBL-producing *Enterobacteriaceae* in different clinical samples in Ethiopia. A meta-analysis was also planned to assess the antibiotic resistance profile of ESBL-producing *Enterobacteriaceae *.

### Risk of bias and sensitivity analysis

The standard error for each original study was calculated using the binomial distribution formula. Evidence for statistical heterogeneity among reported prevalence was using the Cochrane Q-test and I^2^ statistics [28]. The pooled proportion was estimated by using the back-transform of the weighted mean of the transformed proportions for both the fixed-effects model and the random-effects model [29]. A significance level of *P*<0.10 and I^2^>50 % was interpreted as evidence of heterogeneity [30]. A potential source of heterogeneity was investigated by subgroup analysis and meta-regression analysis [31]. Where statistical pooling was not possible, the findings were presented in a narrative form including tables and figures to aid in data presentation where appropriate.

Sensitivity analyses were conducted to determine the relative influence of each individual study on the pooled effect size using a user-written function, metainf. The presence of publication bias was assessed informally by visual inspection of funnel plots [32]. Point prevalence, as well as 95 % confidence intervals, was presented in the forest plot format.

## Results

### Study selection

**Fig. 1.**
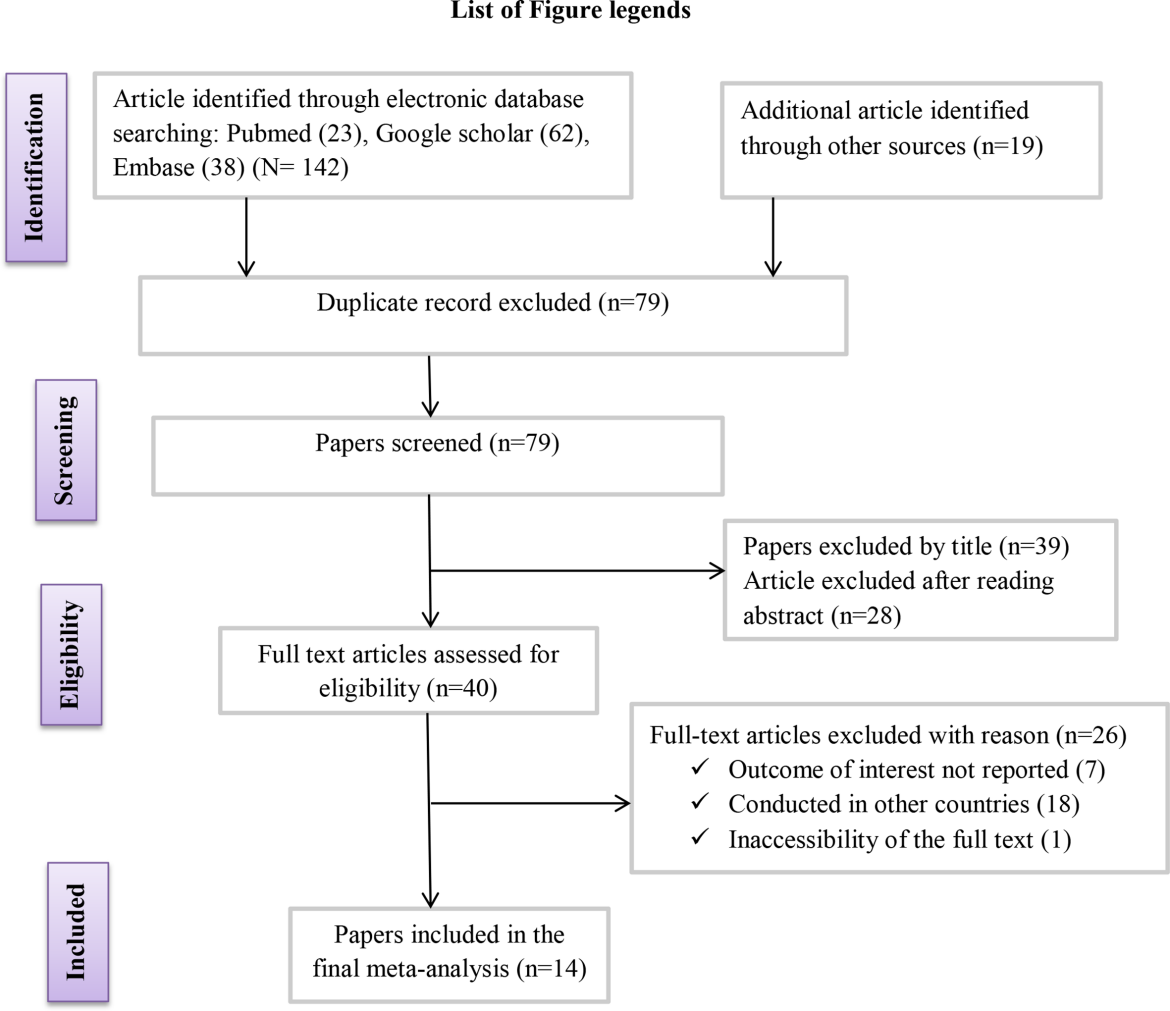
Flow chart of the study selection for systematic review and meta-analysis of the prevalence of ESBL-producing *Enterobacteriaceae* from clinical samples.

The database searches identified a total of 142 articles reporting the prevalence of ESBL-producing *Enterobacteriaceae* in different clinical samples using the range of databases previously described. From these initial articles, 63 were excluded due to duplication. From the remaining 79 articles, 39 were excluded after review of their titles and abstracts confirmed non-relevance to this review, 40 articles were assessed with respect to their eligibility for inclusion, which resulted in the further exclusion of 26 articles primarily due to the study being done in other countries [33–53], and 14 studies were included in the final systematic review and meta-analysis (Fig. 1).

### Description of included studies

In this review, 14 articles published between 2005 and 2019 and that reported the prevalence of ESBL-producing *Enterobacteriaceae* in different clinical specimens were included. In this systematic review and meta-analysis, 2167 *Enterobacteriaceae* isolates were used to determine the pooled prevalence of ESBL-producing *Enterobacteriaceae* in different clinical specimen. The number of *Enterobacteriaceae* isolated in different studies ranged from 57 to 426. The lowest prevalence (16 %) of ESBL-producing *Enterobacteriaceae* was reported in studies conducted in Gondar town [54] and the highest prevalence (79 %) was reported in Addis Ababa [55]. Most of the studies were from Addis Ababa [55–59] and from the Oromia region [57, 60–62] (Table 1).

**Table 1.** Descriptive summary of 14 studies included in the meta-analysis of the prevalence of ESBL-producing *Enterobacteriaceae* in different clinical samples in Ethiopia, 2020

Authors	Year	Study method	Characterization	Study area	* Enterobacteriaceae *	Cases	Prevalence (95 % CI)
Engda *et al*. [54]	2018	Cross-sectional	Phenotypic method	Gondar town	57	9	16 (7–28)
Legese *et al*. [55]	2017	Cross-sectional	Phenotypic method	Addis Ababa	43	34	79 (64–90)
Bitew *et al*. [56]	2019	Cross-sectional	Phenotypic method	Addis Ababa	135	66	49 (40–58)
Abayneh *et al*. [57]	2019	Cross-sectional	Phenotypic method	Jimma town	168	35	21 (15–28)
Beyene *et al*. [58]	2019	Cross-sectional	Phenotypic method	Addis Ababa	238	158	66 (60–72)
Desta *et al*. [59]	2016	Cross-sectional	Phenotypic method	Addis Ababa	295	151	51 (48–82)
Abayneh *et al*. [60]	2018	Cross-sectional	Phenotypic method	Jimma town	74	17	23 (18–35)
Gashaw *et al*. [61]	2018	Cross-sectional	Phenotypic method	Jimma town	100	51	51 (41–61)
Zeynudin *et al*. [62]	2018	Cross-sectional	Genotypic analysis	Jimma town	112	71	63 (54–72)
Teklu *et al*. [86]	2019	Cross-sectional	Phenotypic method	Addis Ababa	426	246	58 (53–62)
Moges *et al*. [84]	2019	Cross-sectional	Phenotypic method	Bahar dar	185	127	69 (61–75)
Solomon *et al*. [87]	2017	Cross-sectional	Phenotypic method	Wolaita Sodo	67	39	58 (46, 70)
Abera *et al*. [73]	2016	Cross-sectional	Phenotypic method	Bahar dar	210	120	57 (50, 63)
Eguale *et al*. [88]	2005	Cross-sectional	Genotypic analysis	Harar	57	19	33 (21, 47)

### Risk of bias

A risk of bias tool [63] was used to assess the risk of bias for the included studies and >90 % of the studies had a low risk of bias. In almost all studies, different clinical samples were collected from patients who attended different health institutions. All *Enterobacteriaceae* were screened for ESBL production using cefotaxime and ceftazidime, and double-disc synergy methods were used for detection of ESBL-producing strains in almost all of the studies.

### Prevalence of ESBL-producing *Enterobacteriaceae* in Ethiopia

**Fig. 2.**
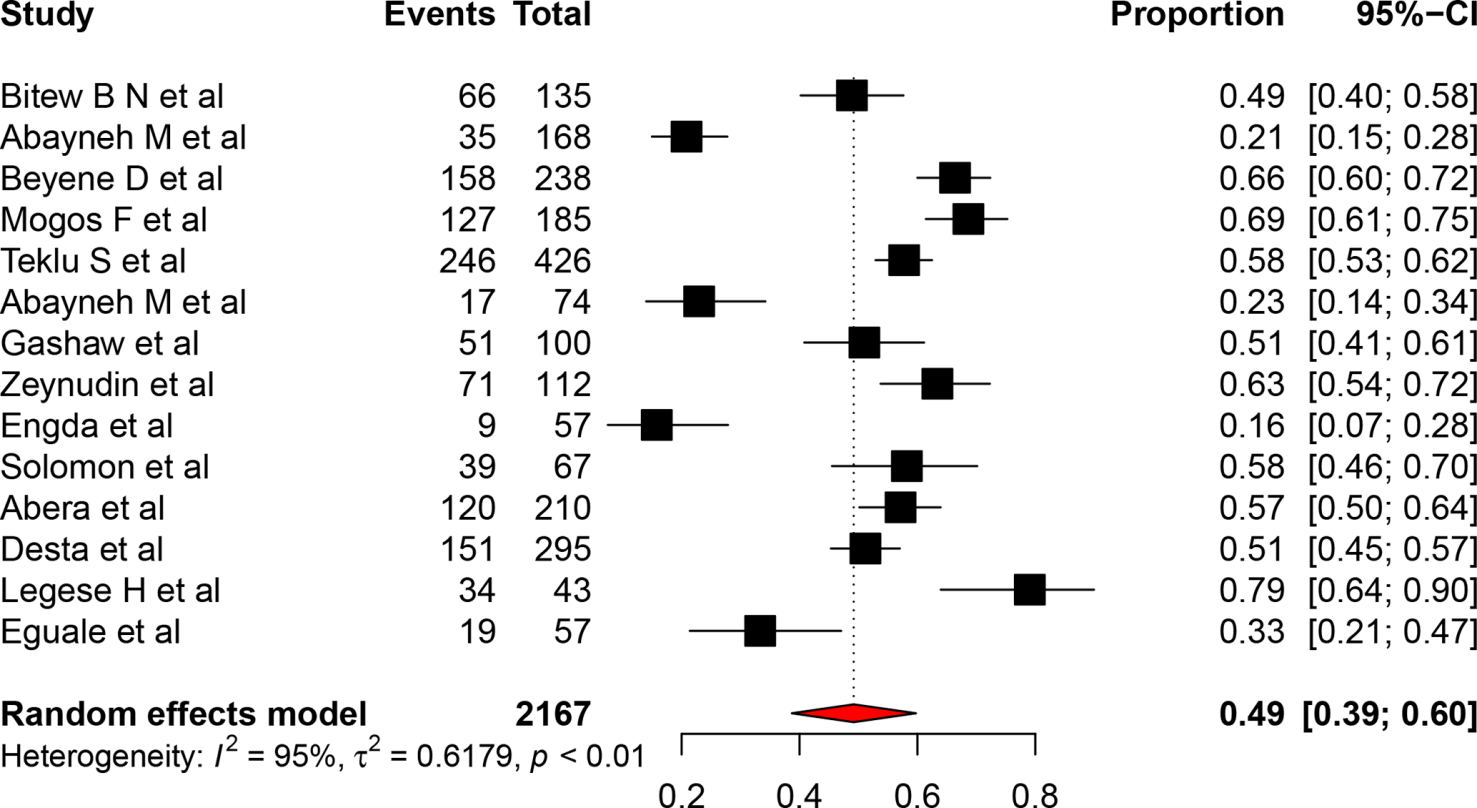
Forest plot of the pooled prevalence of ESBL-producing *Enterobacteriaceae* in different clinical samples in Ethiopia.

The pooled prevalence of ESBL-producing *Enterobacteriaceae* in different clinical specimen in Ethiopia was 49 % (95 % CI: 39, 60). Due to the presence of high heterogeneity (I^2^=62, *P*<0.01), a random effect meta-analysis model was explored to assess the pooled prevalence of *Campylobacter* species in children less than 5 years old in Ethiopia (Fig. 2).

To identify sources of heterogeneity, we assessed the year when the study was published, study area and sample size using univariate meta-regression models, but all were statistically non-significant for the included study (Table 2). A funnel plot showed an irregular distribution of the articles.

**Table 2.** ESBL-producing *Enterobacteriaceae* prevalence in different clinical samples and assessment of source of heterogeneity (based on univariate metaregression) in Ethiopia, 2020

Variable	Coefficient	*P* value
Publication year	0.23	0.27
Sample size	0.60	0.02
**Region**		
Oromia	0.70	0.21
Addis Ababa	0.6	0.30
Amhara	0.07	0.13
SNNPR	na	na
Harar	na	na

SNNPR, Southern Nation Nationality Peoples Region.

### Subgroup analysis

In the current study, subgroup analysis was done based on the area where the study was performed. Based on subgroup analysis, the distribution of ESBL-producing *Enterobacteriaceae* throughout the country varied from region to region. A high prevalence of ESBL-producing Enterobacteriaceae was reported in Addis Ababa followed by SNNPR, with a prevalence of 63.5 and 58 %, respectively (Table 3).

**Table 3.** The magnitude of ESBL-producing *Enterobacteriaceae* in Ethiopia by subgrouping, 2020 (*n*=14)

Variable	Characteristics	Included study	* Enterobacteriaceae* isolated	Prevalence (95 % CI)
Region	Addis Ababa	5	1137	53.8 (54, 72.8)
	Oromia	4	454	39.5 (31, 51.3)
	Amhara	3	452	47.3 (39.3, 55.3)
	Harar	1	57	33 (21, 47)
	SNNPR	1	67	58 (46, 70)
Sample size	>100	9	1869	83.1 (40, 61.4)
	<100	5	298	16.9 (33.3, 58.5)

### The magnitude of ESBL-producing *Enterobacteriaceae* in different clinical samples in different study areas in Ethiopia

**Fig. 3.**
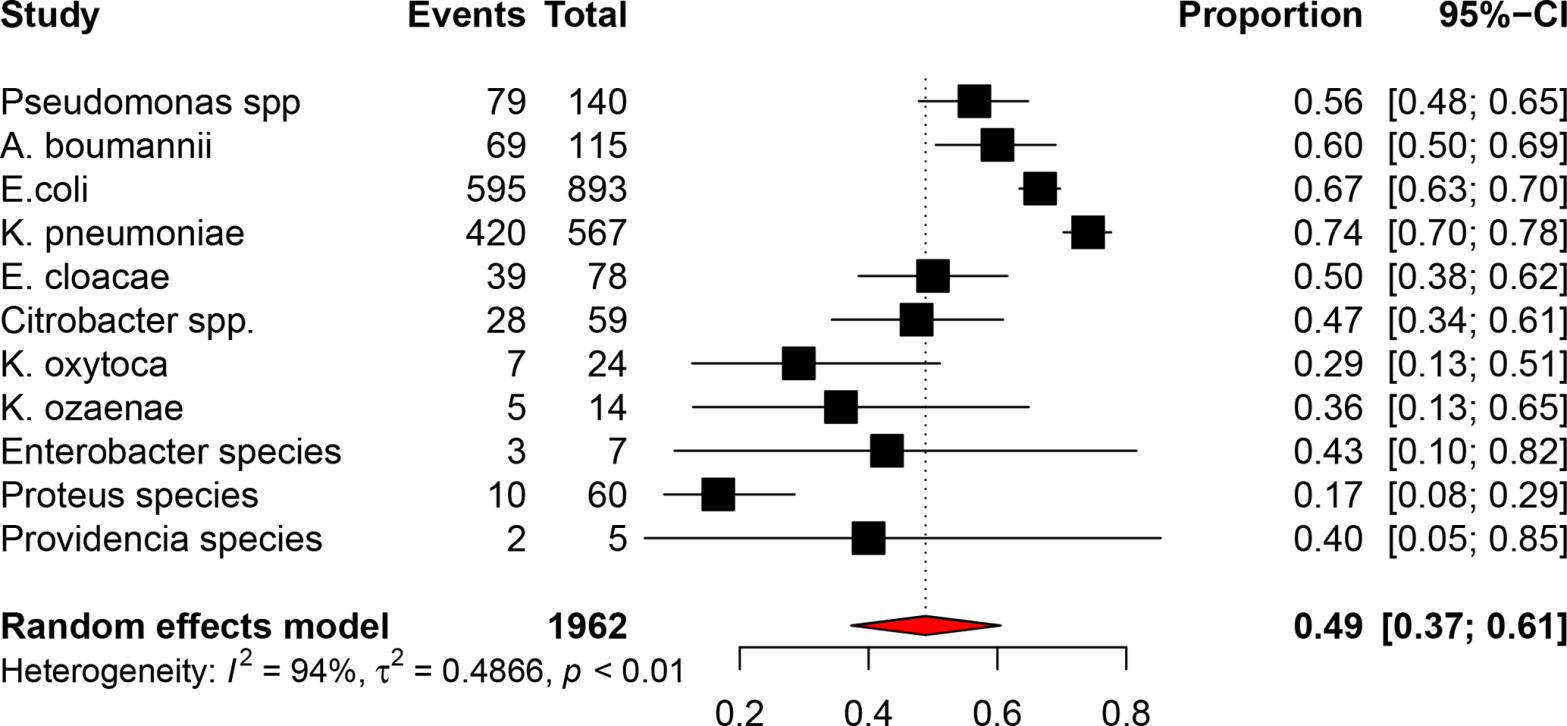
The magnitude of ESBL-producing *Enterobacteriaceae* in different clinical samples in different study areas in Ethiopia, 2020.

In this study, we tried to assess the prevalence of ESBL-producing *Enterobacteriaceae* in different clinical specimen collected from patients. About 1962 *Enterobacteriaceae* among 11 species were collected from 14 studies conducted in Ethiopia. *Klebsiella pneumoniae* was the leading ESBL-producing *Enterobacteriaceae* followed by *Escherichia coli* and *Acinetobacter baumannii* with prevalences of 74, 67 and 60%, respectively. *Proteus* species were the least frequent ESBL-producing *Enterobacteriaceae* with a prevalence of 17 % (Fig. 3).

### Distribution of ESBL-producing *Enterobacteriaceae* in different specimens

In the present study, ESBL-producing *Enterobacteriaceae* were found predominantly in blood specimens (62.3 %), followed by urine specimens (41.2 %) and wounds (35 %). Based on the data collected from the included studies, none of the ESBL-producing *Enterobacteriaceae* was isolated from body fluid, nasal swab or sputum sample (0 %) (Table 4)

**Table 4.** The magnitude of ESBL-producing *Enterobacteriaceae* in different clinical samples collected from different studies conducted in Ethiopia, 2020

Sample no.	Specimen	Number of *Enterobacteriaceae* isolated	ESBL-producing *Enterobacteriaceae *	Prevalence of ESBL-producing *Enterobacteriaceae *
1	Urine	587	242	41.2 %
2	Blood	377	235	62.3 %
3	Wound	120	42	35 %
4	Ear	38	1	2.6 %
5	Body fluid	25	0	0 %
6	Nasal	9	0	0 %
7	Sputum	7	0	0%

### Antibiotic resistance profile of ESBL-producing *Enterobacteriaceae*

In the present systematic review and meta-analysis, ESBL-producing isolates were highly resistant to both third-generation cephalosporins and non-beta lactam antimicrobial agents. Higher resistance rates were recorded among cefotaxime, ceftriaxone, amoxacillin clauvilic acid, ampicillin and aztreonam with values of >90 % (Table 5).

**Table 5.** Pooled prevalence of antibiotic resistance profiles of ESBL-producing *Enterobacteriaceae* to different antibiotics, 2020 R, resistant; S, susceptible.

Antibiotic	Abayneh *et al.* [57] (ESBL=7)		Desta *et al.* [59] (ESBL=151)		Abayneh *et al.* [60] (ESBL=17)		Solomon *et al.* [87] (ESBL=15)		Abera *et al.* [73] (ESBL=120)		Total *n* (%)	
	R	S	R	S	R	S	R	S	R	S	R	S
Cefotaxime	6	1	147	4	17	0	11	4	–	–	181 (95.3)	9 (4.7)
Ceftazidime	5	2	141	10	12	5	11	4	–	–	169 (88.9)	21 (11.1)
Ceftriaxone	6	1	–	–	17	0	13	2	–	–	36 (92.3)	3 (7.7)
AMC	6	1	140	11	14	3	–	–	–	–	160 (91.4)	15 (8.6)
Ampicillin	7	0	–	–	17	0	–	–	–	–	24 (100)	0 (0.0)
Gentamycin	1	6	95	56	11	6	–	–	–	–	107 (61.1)	68 (38.9)
Amikacin	0	7	2	149	4	13	–	–	–	–	6 (3.4)	169 (96.6)
Ciprofloxacin	1	6	117	34	13	4	–	–	69	51	200 (67.8)	95 (32.2)
Tetracycline	6	1	–	–	14	3	–	–	–	–	20 (83.3)	4 (16.7)
SXT	6	1	140	11	14	3	–	–	98	22	258 (87.5)	37 (12.5)
Aztreonam	–	–	147	4	–	–	10	5	–	–	157 (94.6)	9 (5.4)
Impenem	–	–	–	–	0	17	5	10	–	–	5 (15.6)	27 (84.4)
C	–	–	–	–	12	5	–	–	94	26	106(77.4)	31 (22.6)

AMC, amoxicillin-clavulanic acid; C, chloramphicol; SXT, trimethoprim-sulphamethoxazole.

The resistance rates of ESBL-producing isolates to ceftazidime, tetracycline, SXT and chloramphenicol were 77–89 %. However, amikacin and impenem showed greater efficacy against ESBL-producing *Enterobacteriaceae* with efficacy rates of 84–97 % (Table 5).

## Discussion

Data on the magnitude of ESBL-producing *Enterobacteriaceae* in different clinical samples collected from different studies conducted in Ethiopia are limited and are not currently available in aggregated form. The emergence and rapid spread of multidrug resistance strains of ESBL-producing *Enterobacteriaceae* is a serious public health issue worldwide. Rapid expansion of ESBLs is greatly affecting the activity of broad-spectrum antibiotics, creating major therapeutic difficulties with a significant impact on patient outcomes [64].

The phenotypic information obtained in the current meta-analysis indicates a significant prevalence of ESBL producers. The overall magnitude of ESBL-producing *Enterobacteriaceae* in different clinical samples obtained from this study was 49 %, indicating a remarkable health problems in developing and developed countries [65]. Our finding is consistent with studies conducted in Tanzania [66], Nigeria [43], Burkina Faso [67] and Ghana [68] with prevalence ranging from 44 to 58 %. However, it is higher than for studies conducted in Italy [69], Egypt [70] and Turkey [71] with prevalence ranging from 0.5 to 24 % respectively. This variation might be explained by methodological differences, differences in study area and quality of media used. The use of low-quality antibiotics, inappropriate use of antibiotics, and weak infection prevention measures may additionally contribute to the high prevalence of ESBLs.

Based on this meta-analysis, *K. pneumonia* was the leading ESBL-producing *Enterobacteriaceae* with a prevalence of 74 %, followed by *E. coli* (67 %) and *A. baumannii* (60 %). This was in agreement with studies conducted in Uganda [72] with prevalence of *K. pneumoniae* (72.7 %) and *E. coli* (58.1 %) and elsewhere [73] with prevalence of *K. pneumoniae* (69.8 %) and *E. coli* (58.2 %). However, *E. coli* was a predominant ESBL producer compared to *K. pneumoniae* according to studies in Burkina Faso [67] (* E. coli* 67.5 %, *K. pneumoniae* 26 %), India [69] (* E. coli* 61.4 %, *K. pneumoniae* 46.2 %) and Central India [74] (* E. coli* 50.14 %, *K. pneumoniae* 48.27 %). Rapid adaptation to selective changes in environmental pressures, upregulation of the intrinsic resistance mechanisms, and acquisition and transfer of drug resistance genes through mobile genetic elements such as plasmids and transposons could be a possible explanation for an elevated overall drug resistance prevalence rate against different categories of drugs.

In the present study, a high prevalence of ESBL-producing *Enterobacteriaceae* was found in blood specimens (62.3 %), followed by urine specimens (41.2 %) and wounds (35 %). This is in line with studies conducted in Iran [75] (87.8 % in blood, 48.5 % in urine), India [76] (66.67 % in blood, 54.67 % in urine), Burkina Faso [67] (75 % in blood) and north-west India [77] (79.2.0 % in blood). However, studies conducted in Uganda [72] (64.9 % in urine, 47.4 % in pus), Bangladesh [78] (70.4 % in urine, 16.5 % in blood) and central India [79] (52.28 % in urine) reported urine specimens as the major source of ESBL-producing *Enterobacteriaceae *. This indicates that ESBL-producing *Enterobacteriaceae* are becoming a serious problem in the treatment of invasive bacterial infections.

In this meta-analysis, ESBL-producing *Enterobacteriaceae* were highly resistant to ampicillin, followed by cefotaxime, aztreonam, AMC and ceftazidime, with resistance rates ranging from 88 to 100 %, while the lowest resistance was found against amikacin and impenem, 3–16 %. Our finding agrees with studies conducted in Burkina Faso [67], Ghana [68], Saudi Arabia [80], Israel [81], Poland [82] and Sierra Leone [83]. This indicates that ESBL-producing *Enterobacteriaceae* were rapidly emerging in developing countries. In this meta-analysis, ESBL-producing *Enterobacteriaceae* were resistant not only to third-generation cephalosporins but also to other non-*β* lactam group antibiotics. The findings of this meta-analysis showed that amikacin and impenem had better performance against ESBL-producing *Enterobacteriaceae* than other antibiotics, including cephalosporins. Moges and colleagues [84] also reported that amikacin and imipenem performed better in the treatment of ESBL-producing *Enterobacteriaceae *.

The multidrug resistance nature of ESBL-producing *Enterobacteriaceae* may be explained by the fact that they are plasmid-mediated enzymes that carry multiresistance genes by plasmids, transposons and integrons, and also that they are readily transferred to other bacteria through conjugation, transduction or transformation. Those bacteria may not necessarily be of the same species. Bacteria with multiple resistance to antibiotics are now widely distributed in hospitals, are increasingly being isolated from community settings and have become a serious problem throughout the world [85]. This is the most challenging and alarming condition in the management of infectious diseases associated with ESBL-producing *Entrobacteriaceae.*

### Limitations of the study

The articles for this study were limited to the English language. With the study method (most of them were cross-sectional), this can affect the outcome variable by other confounding variables. Additionally, the small sample size could affect the estimated pooled prevalence of ESBL-producing *Enterobacteriaceae *. This meta-analysis represented studies reported from a limited study area, which may reflect under-representation due to the limited number of studies included. The authors of the primary studies did not mention or characterize whether the isolates studied were hospital-acquired or community-based. This may be the source for the outcome of the study. Furthermore, differences in the methods used to characterize the bacterial isolates may also affect the estimated outcome.

## Conclusion

In this meta-analysis, there was a high prevalence of ESBL-producing *Enterobacteriaceae *, which might contribute to the occurrence of multidrug resistance. Most ESBL-producing isolates were found primarily in blood and urine specimens. The most frequent ESBL-producing *Enterobacteriaceae* were *K. pneumoniae *, *E. coli* and *A. baumannii *. ESBL-producing isolates showed a high rate of resistance to cefotaxime, ceftriaxone, ceftazidime, AMC, ampicillin and aztreonam. The best options for the treatment of ESBL-producing *Enterobacteriaceae* are amikacin and impenem. The rise of ESBL-producing *Enterobacteriaceae* requires strict infection prevention and control strategies and strengthening of diagnostic capacity of laboratory professionals for the detection and surveillance of antibiotic resistance.

### Data availability statement

All data relevant to the study are included in the article or have been uploaded as online supplementary information.

## References

[1] Chiu C-W, Li M-C, Ko W-C, Li C-W, Chen P-L, Chang C-M (2015). Clinical impact of gram-negative nonfermenters on adults with community-onset bacteremia in the emergency department. J Microbiol Immunol Infect.

[2] Ventola CL (2015). The antibiotic resistance crisis: Part 1: causes and threats. P T.

[3] Gelband H, Molly Miller P, Pant S, Gandra S, Levinson J (2015). The state of the world’s antibiotics 2015. Wound Healing Southern Africa.

[4] de La Blanchardière A, Dargère S, Guérin F, Daurel C, Saint-Lorant G (2015). Non-carbapenem therapy of urinary tract infections caused by extended-spectrum β-lactamase-producing Enterobacteriaceae. Medecine et maladies Infectieuses.

[5] UDo H, Services H (2013). Antibiotic resistance threats in the United States. Centers for Disease Control and Prevention.

[6] Kakati B, Agarwal S, Gupta S (2015). Emerging issues regarding management of MDR non-fermenting gram negative ventilator associated pneumonia in a rural catering tertiary care hospital. J Med Sci Clin Res.

[7] Nordmann P, Naas T, Poirel L (2011). Global spread of carbapenemase-producing Enterobacteriaceae. Emerg Infect Dis.

[8] Paterson DL (2006). Resistance in gram-negative bacteria: Enterobacteriaceae. Am J Infect Control.

[9] Dogan A, Lasch P, Neuschl C, Millrose MK, Alberts R (2013). Atr-Ftir spectroscopy reveals genomic loci regulating the tissue response in high fat diet fed bxd recombinant inbred mouse strains. BMC genomics.

[10] Aiken AM, Mturi N, Njuguna P, Mohammed S, Berkley JA (2011). Risk and causes of paediatric hospital-acquired bacteraemia in Kilifi district Hospital, Kenya: a prospective cohort study. The Lancet.

[11] Abdallah H, Wintermans B, Reuland E, Koek A, Al Naiemi N (2015). Extended-spectrum β-lactamase-and carbapenemase-producing Enterobacteriaceae isolated from Egyptian patients with suspected blood stream infection. PloS one..

[12] McDonald M, Blondeau JM (2010). Emerging antibiotic resistance in ocular infections and the role of fluoroquinolones. J Cataract Refract Surg.

[13] Organization WH (2014). Antimicrobial Resistance: Global Report on Surveillance.

[14] Duthey B (2013). Priority Medicines for Europe and the World a Public Health Approach to Innovation.

[15] Fournier PE, Richet H, Weinstein RA (2006). The epidemiology and control of Acinetobacter baumannii in health care facilities. Clin Infect Dis.

[16] Bassetti M, Ginocchio F, Mikulska M (2011). New Treatment Options Against Gram-Negative Organisms.

[17] Paterson DL, Bonomo RA (2005). Extended-spectrum β-lactamases: a clinical update. Clinical microbiology reviews.

[18] Pitout JDD, Laupland KB (2008). Extended-Spectrum β-lactamase-producing Enterobacteriaceae: an emerging public-health concern. Lancet Infect Dis.

[19] Alvarez-Uria G, Gandra S, Mandal S, Laxminarayan R (2018). Global forecast of antimicrobial resistance in invasive isolates of *Escherichia coli* and Klebsiella pneumoniae. Int J Infect Dis.

[20] Murray TS, Peaper DR (2015). The contribution of extended-spectrum β-lactamases to multidrug-resistant infections in children. Curr Opin Pediatr.

[21] Bevan ER, Jones AM, Hawkey PM (2017). Global epidemiology of CTX-M β-lactamases: temporal and geographical shifts in genotype. J Antimicrob Chemother.

[22] Bush K, Jacoby GA (2010). Updated functional classification of β-lactamases. Antimicrob Agents Chemother.

[23] Arksey H, O'Malley L (2005). Scoping studies: towards a methodological framework. Int J Soc Res Methodol.

[24] Liberati A, Altman DG, Tetzlaff J, Mulrow C, Gøtzsche PC (2009). The PRISMA statement for reporting systematic reviews and meta-analyses of studies that evaluate health care interventions: explanation and elaboration. PLoS medicine..

[25] Moher D, Pham B, Lawson M, Klassen T (2003). The inclusion of reports of randomised trials published in languages other than English in systematic reviews. Health Technol Assess.

[26] Armstrong R, Waters E, Jackson N (2007). Systematic Reviews of Health Promotion and Public Health Interventions.

[27] Cheng Z, Lu Y, Cao Q, Qin L, Pan Z (2020). Clinical features and chest CT manifestations of coronavirus disease 2019 (COVID-19) in a single-center study in Shanghai, China. Am J Roentgenol.

[28] Rücker G, Schwarzer G, Carpenter JR, Schumacher M (2008). Undue reliance on I 2 in assessing heterogeneity may mislead. BMC medical research methodology.

[29] Nyaga VN, Arbyn M, Aerts M (2014). Metaprop: a Stata command to perform meta-analysis of binomial data. Arch Public Health.

[30] Thompson SG, Sharp SJ (1999). Explaining heterogeneity in meta‐analysis: a comparison of methods. Stat Med.

[31] Cochran WG (1950). The comparison of percentages in matched samples. Biometrika.

[32] Egger M, Smith GD, Schneider M, Minder C (1997). Bias in meta-analysis detected by a simple, graphical test. Bmj.

[33] Sharma M, Pathak S, Srivastava P (2013). Prevalence and antibiogram of extended spectrum β-lactamase (ESBL) producing gram negative bacilli and further molecular characterization of ESBL producing *Escherichia coli* and *Klebsiella* spp. J Clin Diagn Res.

[34] Osthoff M, McGuinness SL, Wagen AZ, Eisen DP (2015). Urinary tract infections due to extended-spectrum beta-lactamase-producing gram-negative bacteria: identification of risk factors and outcome predictors in an Australian tertiary referral hospital. Int J Infect Dis.

[35] Shrestha A, Bajracharya AM, Subedi H, Turha RS, Kafle S (2017). Multi-drug resistance and extended spectrum beta lactamase producing gram negative bacteria from chicken meat in Bharatpur metropolitan, Nepal. BMC research notes..

[36] Farzana R, Shamsuzzaman SM, Mamun KZ, Shears P (2013). Antimicrobial susceptibility pattern of extended spectrum beta-lactamase producing gram-negative bacteria isolated from wound and urine in a tertiary care Hospital, Dhaka City, Bangladesh. Southeast Asian J Trop Med Public Health.

[37] Fong JJ, Rosé L, Radigan EA (2012). Clinical outcomes with ertapenem as a first-line treatment option of infections caused by extended-spectrum β-lactamase producing gram-negative bacteria. Ann Pharmacother.

[38] Nasa P, Juneja D, Singh O, Dang R, Singh A (2012). An observational study on bloodstream extended-spectrum beta-lactamase infection in critical care unit: incidence, risk factors and its impact on outcome. Eur J Intern Med.

[39] Dandachi I, Fayad E, El-Bazzal B, Daoud Z, Rolain J-M (2019). Prevalence of extended-spectrum beta-lactamase-producing gram-negative bacilli and emergence of mcr-1 colistin resistance gene in Lebanese swine farms. Microb Drug Resist.

[40] Leylabadlo HE, Pourlak T, Bialvaei AZ, Aghazadeh M, Asgharzadeh M, Kafil HS (2017). Extended-Spectrum beta-lactamase producing gram negative bacteria in Iran: a review. Afr J Infect Dis.

[41] Shaikh S, Fatima J, Shakil S, Danish Rizvi SM, Kamal MA (2015). Prevalence of multidrug resistant and extended spectrum beta-lactamase producing Pseudomonas aeruginosa in a tertiary care hospital. Saudi J Biol Sci.

[42] Doi Y, Adams-Haduch JM, Peleg AY, D'Agata EMC (2012). The role of horizontal gene transfer in the dissemination of extended-spectrum beta-lactamase-producing *Escherichia coli* and *Klebsiella pneumoniae* isolates in an endemic setting. Diagn Microbiol Infect Dis.

[43] Ogefere HO, Aigbiremwen PA, Omoregie R (2015). Extended-spectrum beta-lactamase (ESBL)–producing Gram-negative isolates from urine and wound specimens in a tertiary health facility in southern Nigeria. Trop J Pharm Res.

[44] Rezai MS, Salehifar E, Rafiei A, Langaee T, Rafati M (2015). Characterization of multidrug resistant extended-spectrum beta-lactamase-producing *Escherichia coli* among uropathogens of pediatrics in North of Iran. Biomed Res Int.

[45] Perez F, Bonomo RA (2015). Editorial commentary: bloodstream infection caused by extended-spectrum β-Lactamase–Producing gram-negative bacteria: how to define the best treatment regimen?. Oxford University Press.

[46] Degnan LA, Milstone AM, Diener-West M, Lee CKK (2015). Extended-Spectrum beta-lactamase bacteria from urine isolates in children. J Pediatr Pharmacol Ther.

[47] Tschudin-Sutter S, Frei R, Dangel M, Stranden A, Widmer AF (2012). Rate of transmission of extended-spectrum beta-lactamase-producing Enterobacteriaceae without contact isolation. Clin Infect Dis.

[48] Upadhyay S, Joshi SR (2015). TEM mediated extended spectrum cephalosporin resistance in clinical & environmental isolates of Gram negative bacilli: A report from northeast India. Indian J Med Res.

[49] Kang C-I, Chung DR, Ko KS, Peck KR, Song J-H (2012). Risk factors for infection and treatment outcome of extended-spectrum β-lactamase-producing *Escherichia coli* and *Klebsiella pneumoniae* bacteremia in patients with hematologic malignancy. Ann Hematol.

[50] Zaniani FR, Meshkat Z, Naderi Nasab M, Khaje-Karamadini M, Ghazvini K (2012). The prevalence of TEM and SHV genes among extended-spectrum beta-lactamases producing *Escherichia coli* and *Klebsiella pneumoniae*. Iran J Basic Med Sci.

[51] Tham J, Walder M, Melander E, Odenholt I (2012). Prevalence of extended-spectrum beta-lactamase-producing bacteria in food. Infect Drug Resist.

[52] Saeidi S, Amini Boroujeni N, Ahmadi H, Hassanshahian M (2015). Antibacterial activity of some plant extracts against extended- spectrum beta-lactamase producing Escherichia coli isolates. Jundishapur J Microbiol.

[53] Oli AN, Eze DE, Gugu TH, Ezeobi I, Maduagwu UN (2017). Multi-antibiotic resistant extended-spectrum beta-lactamase producing bacteria pose a challenge to the effective treatment of wound and skin infections. Pan Afr Med J.

[54] Engda T, Moges F, Gelaw A, Eshete S, Mekonnen F (2018). Prevalence and antimicrobial susceptibility patterns of extended spectrum beta-lactamase producing Entrobacteriaceae in the University of Gondar referral hospital environments, Northwest Ethiopia. BMC Res Notes.

[55] Legese MH, Weldearegay GM, Asrat D (2017). Extended-spectrum beta-lactamase- and carbapenemase-producing *Enterobacteriaceae* among Ethiopian children. Infect Drug Resist.

[56] Bitew A (2019). High prevalence of multi-drug resistance and extended spectrum beta lactamase production in non-fermenting gram-negative bacilli in Ethiopia. Infect Dis Res Treat.

[57] Abayneh M, Tesfaw G, Woldemichael K, Yohannis M, Abdissa A (2019). Assessment of extended-spectrum β-lactamase (ESBLs)–producing *Escherichia coli* from minced meat of cattle and swab samples and hygienic status of meat retailer shops in Jimma town, Southwest Ethiopia. BMC Infect Dis.

[58] Beyene D, Bitew A, Fantew S, Mihret A, Evans M (2019). Multidrug-resistant profile and prevalence of extended spectrum β-lactamase and carbapenemase production in fermentative Gram-negative bacilli recovered from patients and specimens referred to national reference laboratory, Addis Ababa, Ethiopia. PloS one.

[59] Desta K, Woldeamanuel Y, Azazh A, Mohammod H, Desalegn D (2016). High gastrointestinal colonization rate with extended-spectrum β-lactamase-producing Enterobacteriaceae in hospitalized patients: emergence of carbapenemase-producing *K. pneumoniae* in Ethiopia. PloS one.

[60] Abayneh M, Tesfaw G, Abdissa A (2018). Isolation of extended-spectrum *β*-lactamase- (ESBL-) producing *Escherichia coli* and *Klebsiella pneumoniae* from patients with community-onset urinary tract infections in Jimma University Specialized Hospital, Southwest Ethiopia. Can J Infect Dis Med Microbiol.

[61] Gashaw M, Berhane M, Bekele S, Kibru G, Teshager L (2018). Emergence of high drug resistant bacterial isolates from patients with health care associated infections at Jimma University medical center: a cross sectional study. Antimicrob Resist Infect Control.

[62] Zeynudin A, Pritsch M, Schubert S, Messerer M, Liegl G (2018). Prevalence and antibiotic susceptibility pattern of CTX-M type extended-spectrum β-lactamases among clinical isolates of gram-negative bacilli in Jimma, Ethiopia. BMC infectious diseases.

[63] Hoy D, Brooks P, Woolf A, Blyth F, March L (2012). Assessing risk of bias in prevalence studies: modification of an existing tool and evidence of interrater agreement. J Clin Epidemiol.

[64] Adler A, Katz DE, Marchaim D (2016). The continuing plague of extended-spectrum β-lactamase–producing Enterobacteriaceae infections. Infectious Disease Clinics.

[65] Habeeb MA, Sarwar Y, Ali A, Salman M, Haque A (2013). Rapid emergence of ESBL producers in *E. coli* causing urinary and wound infections in Pakistan. Pakistan journal of medical sciences.

[66] Moyo SJ, Aboud S, Kasubi M, Lyamuya EF, Maselle SY (2010). Antimicrobial resistance among producers and non-producers of extended spectrum beta-lactamases in urinary isolates at a tertiary hospital in Tanzania. BMC Res Notes.

[67] Ouedraogo A-S, Sanou M, Kissou A, Sanou S, Solaré H (2016). High prevalence of extended-spectrum ß-lactamase producing Enterobacteriaceae among clinical isolates in Burkina Faso. BMC infectious diseases.

[68] Obeng-Nkrumah N, Twum-Danso K, Krogfelt KA, Newman MJ (2013). High levels of extended-spectrum beta-lactamases in a major teaching hospital in Ghana: the need for regular monitoring and evaluation of antibiotic resistance. Am J Trop Med Hyg.

[69] Rao SP, Rama PS, Gurushanthappa V, Manipura R, Srinivasan K (2014). Extended-Spectrum beta-lactamases producing *Escherichia coli* and *Klebsiella pneumoniae*: a multi-centric study across Karnataka. J Lab Physicians.

[70] Fam N, Leflon-Guibout V, Fouad S, Aboul-Fadl L, Marcon E (2011). CTX-M-15-producing *Escherichia coli* clinical isolates in Cairo (Egypt), including isolates of clonal complex ST10 and clones ST131, ST73, and ST405 in both community and hospital settings. Microbial Drug Resistance.

[71] Nijssen S, Florijn A, Bonten MJM, Schmitz FJ, Verhoef J (2004). Beta-Lactam susceptibilities and prevalence of ESBL-producing isolates among more than 5000 European Enterobacteriaceae isolates. Int J Antimicrob Agents.

[72] Kateregga JN, Kantume R, Atuhaire C, Lubowa MN, Ndukui JG (2015). Phenotypic expression and prevalence of ESBL-producing Enterobacteriaceae in samples collected from patients in various wards of Mulago Hospital, Uganda. BMC Pharmacol Toxicol.

[73] Abera B, Kibret M, Mulu W (2016). Extended-Spectrum beta (β)-lactamases and Antibiogram in Enterobacteriaceae from clinical and drinking water Sources from Bahir Dar City, Ethiopia. PloS one.

[74] Alfola M, Kamel Z, Nada M, Rashed LA, El-Awady BA (2017). Phenotypic and Genotypic Characterization of ESBL-Producing *Escherichia coli* and *Klebsiella pneumonia* isolates from Patient’s Urine specimens. Int Arabic J Antimicrob Agents.

[75] Mansouri S, Abbasi S (2010). Prevalence of multiple drug resistant clinical isolates of extended-spectrum beta-lactamase producing Enterobacteriaceae in Southeast Iran.

[76] Kumar D, Singh AK, Ali MR, Chander Y (2014). Antimicrobial susceptibility profile of extended spectrum β-lactamase (ESBL) producing *Escherichia coli* from various clinical samples. Infect Dis.

[77] Hooja S, Pal N, Karadiya R, Sharma R, Mishra R (2016). Prevalence and antimicrobial susceptibility of extended spectrum β-lactamases (ESBL) producing *Escherichia coli* and *Klebsiella pneumoniae* isolates in a tertiary care hospital in north-west India. Int J Curr Microbiol App Sci.

[78] Alipourfard I, Nili NY (2010). Antibiogram of extended spectrum beta-lactamase (ESBL) producing *Escherichia coli* and *Klebsiella pneumoniae* isolated from hospital samples. Bangladesh J Med Microbiol.

[79] Özadam A, Özpinar H (2016). Phenotypic determination of ESBL-and Ampc-producing Enterobacteriaceae in cheese samples. Int J Food Eng Res.

[80] Alyamani EJ, Khiyami AM, Booq RY, Majrashi MA, Bahwerth FS (2017). The occurrence of ESBL-producing Escherichia coli carrying aminoglycoside resistance genes in urinary tract infections in Saudi Arabia. Ann Clin Microbiol Antimicrob.

[81] Schwaber MJ, Navon-Venezia S, Schwartz D, Carmeli Y (2005). High levels of antimicrobial coresistance among extended-spectrum-beta-lactamase-producing Enterobacteriaceae. Antimicrob Agents Chemother.

[82] Esiobu N, Armenta L, Ike J (2002). Antibiotic resistance in soil and water environments. Int J Environ Health Res.

[83] Leski TA, Taitt CR, Bangura U, Stockelman MG, Ansumana R (2016). High prevalence of multidrug resistant Enterobacteriaceae isolated from outpatient urine samples but not the hospital environment in bo, Sierra Leone. BMC infectious diseases.

[84] Moges F, Setegn Eshetie WA, Mekonnen F, Dagnew M, Endale A (2019). High prevalence of extended-spectrum beta-lactamase-producing gram-negative pathogens from patients attending Felege Hiwot comprehensive specialized Hospital, Bahir Dar, Amhara region. PloS one.

[85] Jacoby GA, Medeiros AA (1991). More extended-spectrum beta-lactamases. Antimicrobial Agents and Chemotherapy.

[86] Teklu DS, Negeri AA, Legese MH, Bedada TL, Woldemariam HK (2019). Extended-spectrum beta-lactamase production and multi-drug resistance among *Enterobacteriaceae* isolated in Addis Ababa, Ethiopia. Antimicrob Resist Infect Control.

[87] Solomon FB, Wadilo F, Tufa EG, Mitiku M (2017). Extended spectrum and metalo beta-lactamase producing airborne *Pseudomonas aeruginosa* and *Acinetobacter baumanii* in restricted settings of a referral hospital: a neglected condition. Antimicrob Resist Infect Control.

[88] Eguale T, Birungi J, Asrat D, Njahira MN, Njuguna J (2017). Genetic markers associated with resistance to beta-lactam and quinolone antimicrobials in non-typhoidal *Salmonella* isolates from humans and animals in central Ethiopia. Antimicrob Resist Infect Control.

